# The Role of Seizure-Related *SEZ6* as a Susceptibility Gene in Febrile Seizures

**DOI:** 10.1155/2011/917565

**Published:** 2011-07-16

**Authors:** John C. Mulley, Xenia Iona, Bree Hodgson, Sarah E. Heron, Samuel F. Berkovic, Ingrid E. Scheffer, Leanne M. Dibbens

**Affiliations:** ^1^Department of Genetic Medicine, Directorate of Genetics and Molecular Pathology, SA Pathology at Women's and Children's Hospital, Adelaide, SA 5006, Australia; ^2^School of Molecular and Biomedical Sciences, Discipline of Genetics, The University of Adelaide, Adelaide, SA 5000, Australia; ^3^School of Pediatrics and Reproductive Health, Discipline of Pediatrics, The University of Adelaide, Adelaide, SA 5000, Australia; ^4^School of Pharmacy and Medical Sciences, University of South Australia, Adelaide, SA 5000, Australia; ^5^Epilepsy Research Centre, University of Melbourne (Austin Health), West Heidelberg, VIC 3081, Australia; ^6^Department of Paediatrics, University of Melbourne, Royal Children's Hospital, Melbourne, VIC 3010, Australia; ^7^Epilepsy Research Program, University of South Australia, City East Campus, C5-45, Adelaide, SA 5000, Australia

## Abstract

Sixty cases of febrile seizures from a Chinese cohort had previously been reported with a strong association between variants in the seizure-related (*SEZ*) 6 gene and febrile seizures. They found a striking lack of genetic variation in their controls. We found genetic variation in *SEZ6* at similar levels at the same DNA sequence positions in our 94 febrile seizure cases as in our 96 unaffected controls. Two of our febrile seizure cases carried rare variants predicted to have damaging consequences. Combined with some of the variants from the Chinese cohort, these data are compatible with a role for *SEZ6* as a susceptibility gene for febrile seizures. However, the polygenic determinants underlying most cases of febrile seizures with complex inheritance remain to be determined.

## 1. Introduction

 Febrile seizures affect 3% of infants between the ages of three months and five years of age and are associated with fever in the absence of intracranial infection or other defined cause. Febrile seizures are generally thought to be multifactorial with the genetic component polygenic, as suggested by diminishing risks beyond first degree relatives. As with other genetic forms of epilepsy, there are rare families with presumed autosomal dominant inheritance of febrile seizures [1, 2]. These may lead to gene identification potentially offering leads to genes and gene families that may harbour susceptibility variants for the vast majority of febrile seizures with complex inheritance.

 Seizure-related (SEZ) 6 is a protein of 994 amino acids which is thought to play a role in neuronal cell to cell signalling. Normal functioning maintains a balance between dendrite growth and branching to optimize dendritic trees for synaptic connectivity [3]. *SEZ6* was originally cloned following upregulation in mouse neurons after seizure induction using pentylenetetrazole (PTZ) stimulation [4]. This raises the question: can disturbances in the amount of transcribed *SEZ6* through naturally occurring mutations predispose to seizures? Yu et al. [5] subsequently reported a strong association between *SEZ6* and human febrile seizures indicating that *SEZ6* is a susceptibility gene for febrile seizures with complex inheritance.

## 2. Methods

 Ninety-four (47 males, 47 females) unrelated cases of simple febrile seizure were screened for *SEZ6* mutations in genomic DNA isolated from venous blood. Diagnosis was based on the observation of a seizure which ceased within approximately three minutes and which did not recur within a 24-hour period. For all cases, seizure onset occurred after three months of age and had ceased in all subjects within five years of age. Ninety-six anonymous blood donors from the same Caucasian population were used as controls. None of the affected children required lumbar puncture, electroencephalograhy, blood studies, or neuroimaging, consistent with the management recommendations of Duffner et al. [6]. 

The 17 *SEZ6* exons were PCR amplified using the flanking intronic primers listed in Table 1. Primers were designed based on the sequence of *SEZ6* transcript variant 1 (NCBI Accession Number NM_178860). Samples were screened by single-stranded conformation polymorphism analysis (SSCA) using the GelScan 3000 (Corbett Research) according to the manufacturer's instructions. The functional effects of nonsynonymous variants were predicted using the PolyPhen-2 tool (http://genetics.bwh.harvard.edu/pph2/). 

## 3. Results

**Figure 1:**
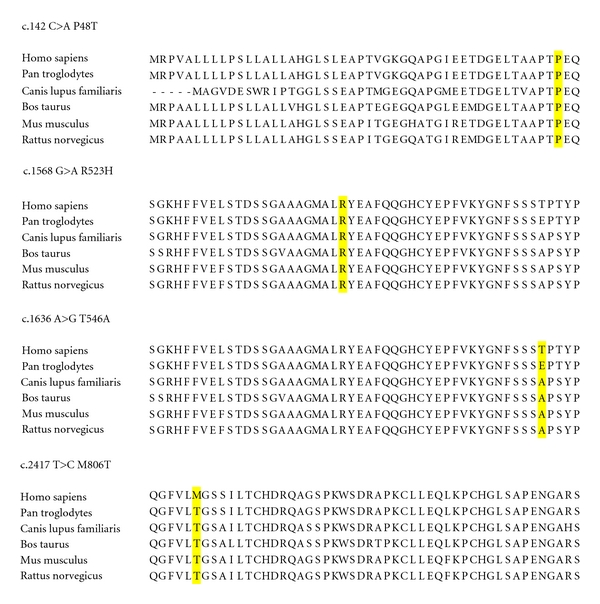
Evolutionary conservation of amino acid positions across vertebrates at sites of rare and common nonsynonymous amino acid substitutions within the SEZ6 protein.

 Nine sequence variants were detected within the protein-coding regions of the *SEZ6 *gene (Table 2). Five of the changes were synonymous and did not change the amino acid, but four of the changes were nonsynonymous and altered the amino acid. Two of the missense changes, in exon 2 (c.142 C>A; p.P48T) and exon 7 (c.1568 G>A; p.R523H), are both low-frequency changes but are present in cases of febrile seizures and in controls. They each affect amino acids which are highly conserved across vertebrate species (Figure 1). Both of these changes were predicted by PolyPhen-2 to be damaging, with scores of 0.955 and 1.000, respectively. Neither change has been reported as a known variant in dbSNP (http://www.ncbi.nlm.nih.gov/snp/). 

 The remaining two missense changes, the exon 8 c.1636 A>G (causing p.T546A) and exon 12 c.2417 T>C (causing p.M806T), were common, occurring with approximately equal frequency in both febrile seizure cases and controls. They are moderately conserved across vertebrates. Both of these changes are listed in dbSNP (rs1976165 and rs12941884, resp.) with frequencies in the European population similar to those seen in our patient and control groups. These two changes were predicted to be benign by PolyPhen-2 analysis. Allele frequencies affecting all coding regions did not markedly differ between febrile seizures (*N* = 94) and controls (*N* = 96). There was no evidence in our population, from the sample size examined, for an association of the strength previously reported between *SEZ6* genetic variation and febrile seizures. We also detected an insertion in intron 5 of the *SEZ6* gene (IVS5+10-11insC) with allele frequencies of 51.6% in patients and 57.3% in controls. This change is intronic and likely benign.

## 4. Discussion

 The most common of the *SEZ6* variants in the population studied is the IVS5+10-11insC. This is the same variant reported by Yu et al. [5] as occurring in exon 5 at their position 1435 in the cDNA (their GenBank accession number GI:20143984). The discrepancy between the position they report for this polymorphism and the position we report arises from differences in the cDNA sequences used. In both isoforms of *SEZ6* currently listed in GenBank (NM_178860 and NM_001098635), the variant is intronic. It is likely that this variant is a benign polymorphism, as evidenced by its high frequency among both cases and controls in our study (Table 2). The variant is also listed in dbSNP (rs58747412), but the entry does not include population frequency data. 

 Critical examination of the data reported by Yu et al. [5] reveals that in addition to the intronic variant described above, a threonine to alanine missense variant T546A, observed in four of their patients is in fact the SNP rs1976165, which we observed at similar frequency in both patients and controls.

 Contrary to the report of Yu et al. we detected the same degree of genetic variation in both the febrile seizure cases and our 96 controls. Their control numbers were not of adequate size, and in their febrile seizure cases, they misinterpreted the presence of naturally occurring genetic variations to be multiple pathogenic mutations present only among the febrile seizure patients. 

 Data from dbSNP shows that *SEZ6* is a highly variable gene, with 17 nonsynonymous coding SNPs listed. Findings of variants in this gene, therefore, need to be interpreted with caution in the absence of additional data (such as protein alignments, *in silico *pathogenicity predictions, or functional studies) indicating that the variants are indeed deleterious. PolyPhen-2 predictions indicate that only three or perhaps four of the eight coding variants reported by Yu et al. are likely to be deleterious. These and our data reporting two additional potentially damaging rare variants are suggestive of a contribution of *SEZ6* to a genetic predisposition to FS in a proportion of cases.

 The extent of the evolutionary conservation for some of the variants detected warrants further investigation using much larger sample sizes. Massively parallel sequencing (MPS) [7] now delivers the sensitivity to detect all rare variants. The focus needs to shift to rare variants [8] and statistical developments enabling association tests on rare variants [9]. The polygenic heterogeneity model [10, 11] is likely applicable to febrile seizures as well as for genetic generalised epilepsy. It predicts that rare variants conferring genetic susceptibility will be present in both cases and controls; however, their presence throughout the gene will be significantly higher in cases than in controls if large and adequately powered sample sizes are analysed for the true susceptibility genes.

## 5. Conclusion

 Taken together, portions of the data from the present study and from Yu et al. [5] suggest a role for *SEZ6* as a susceptibility gene for febrile seizures. There is no reason to restrict *SEZ6* analyses to febrile seizures since this gene represents a plausible candidate for any seizure disorder based on how it was originally cloned [4]. A significant proportion of febrile seizure cases progress to afebrile seizures [12] suggesting shared genetic determinants between febrile and a febrile seizures. *SEZ6* warrants further investigation as a susceptibility gene for both febrile seizures and the epilepsies which show complex inheritance.

## Figures and Tables

**Table 1:** Primers used to amplify *SEZ6* exons for mutation screening.

*SEZ6*-1F	CGT GGT GCT GAT TCT GTC AG
*SEZ6*-1R	TTG GAC TGG GCA GCC AGA TG
*SEZ6*-2.1F	TGT AGT TCC GTG ATT CTC AGC
*SEZ6*-2.1R	TGG CCT CAG CTC CTC ATC TC
*SEZ6*-2.2F	TTG AAG CTG CTC AAC CAC CAC
*SEZ6*-2.2R	ATG TCT CCA GGA CCC TCT TG
*SEZ6*-2.3F	CTA TGC TTC GAA TCA CAG CTC
*SEZ6*-2.3R	TGG CAC AGT GTC AGA GAC AG
*SEZ6*-3F	TTA GTT GGA CCA CTT CAC CAG
*SEZ6*-3R	ACA TCC TCT CAT AGC ATG TG
*SEZ6*-4F	GCA GCA GGA AGA AGT CTG AC
*SEZ6*-4R	TGC TCC TTC CCT CTA GGA TG
*SEZ6*-5F	CTG CAC ACA TAC ACA GTG TC
*SEZ6*-5R	AAA GTG GCA GAG AGC AAC AG
*SEZ6*-6F	ATA GGG AGG AAG GCA TGT TAG
*SEZ6*-6R	TCC CTC CAG CAG GGT ATT AC
*SEZ6*-7F	TAA TAC CCT GCT GGA GGG AC
*SEZ6*-7R	GTG TGG GAG AAA GAC CCT AG
*SEZ6*-8F	GCC CAC TGT GTT TAATAC CAG
*SEZ6*-8R	TAT TCT CCT GGT ATG ACC CTG
*SEZ6*-9F	AGC AAC ACC ATG GTA AGC TTG
*SEZ6*-9R	TGG GCT GGA CAA GGG ATA TC
*SEZ6*-10F	TAT CCC TTG TCC AGC CCA TC
*SEZ6*-10R	TTG CCA TGG CTT GCT GTC TG
*SEZ6*-11F	GGA CAG TCA CTT TGG TGC TG
*SEZ6*-11R	TCC AGG AGA GAG GTT TGG AG
*SEZ6*-12F	ATT GGC CTC TGC TTA GTT CTC
*SEZ6*-12R	AGT GCA GTG AGG GTG TCA TG
*SEZ6*-13F	GGA GGG AAA ACC TCT AGC TG
*SEZ6*-13R	CAT TGG ACA TCT TTG CCA GAG
*SEZ6*-14F	CTC CCT GCC TTA GTG GTT TG
*SEZ6*-14R	CTC TCT CTT TCT CTG CCC TC
*SEZ6*-15F	GGC AGA GGT GAG AGA ATA TG
*SEZ6*-15R	TGA GGT ATG CAG GTA TGC AG
*SEZ6*-16F	TGC ATA CCT GCA TAC CTC AC
*SEZ6*-16R	AGC AAA GAA CTG GGT CTT GG
*SEZ6*-17F	GGA AGG TGA ATT ATG GCC TG
*SEZ6*-17R	GTT CTT CCC ACA GGT AGA TG

**Table 2:** Genetic variation detected within *SEZ6*.

Amplicon/exon	Position	Amino acid change	Febrile seizure frequency (%) *N* = 94	Control frequency (%) *N* = 96
Rare nonsynonymous missense substitutions				

2.1	c.142 C>A	P48T	0.5	0.5
7	c.1568 G>A	R523H	1.1	0.5

Common nonsynonymous missense substitutions				

8	c.1636 A>G	*T546A	22.4	17.6
12	c.2417 T>C	**M806T	10.6	15.6

Rare synonymous missense substitutions (benign)				

2.1	c.213 G>A	P71P	0.5	0.0
2.2	c.384 G>A	A128A	0.5	0.0
5	c.1209 C>T	P403P	0.5	0.5
7	c.1557 C>T	G519G	0.5	0.5

Common synonymous missense substitutions (benign)				

8	c.1737 C>T	D579D	21.3	17.0

Common intronic insertion				

5	IVS5+10-11insC		51.6	57.3

*rs1976165; **rs12941884.

Numbering of variants in this table is based on the mRNA sequence for *SEZ6* transcript variant 1 (NM_178860), with the A of the initiation codon numbered as nucleotide 1.
